# Is the future of personalized therapy in triple-negative breast cancer based on molecular subtype?

**DOI:** 10.18632/oncotarget.3849

**Published:** 2015-05-07

**Authors:** Fanny Le Du, Bedrich L. Eckhardt, Bora Lim, Jennifer K. Litton, Stacy Moulder, Funda Meric-Bernstam, Ana M. Gonzalez-Angulo, Naoto T. Ueno

**Affiliations:** ^1^ Department of Breast Medical Oncology, The University of Texas MD Anderson Cancer Center, Houston, TX, USA; ^2^ Department of Medical Oncology, Eugène Marquis Cancer Center, Rennes, France; ^3^ Clinical Cancer Genetics Program, The University of Texas Graduate School of Biomedical Sciences, Houston, TX, USA; ^4^ Department of Investigational Cancer Therapeutics, The University of Texas MD Anderson Cancer Center, Houston, TX, USA; ^5^ Department of Systems Biology, The University of Texas MD Anderson Cancer Center, Houston, TX, USA; ^6^ The University of Texas MD Anderson Women's Cancer Moons Shot Program, Houston, TX, USA

**Keywords:** triple-negative breast cancer, gene expression profiling, epithelial-mesenchymal transition, immunotherapy, targeted therapy

## Abstract

Significant research has been conducted to better understand the extensive, heterogeneous molecular features of triple-negative breast cancer (TNBC). We reviewed published TNBC molecular classifications to identify major groupings that have potential for clinical trial development. With the ultimate aim to streamline translational medicine, we linked these categories of TNBC according to their gene-expression signatures, biological function, and clinical outcome. To this end, we define five potential clinically actionable groupings of TNBC: 1) basal-like TNBC with DNA-repair deficiency or growth factor pathways; 2) mesenchymal-like TNBC with epithelial-to-mesenchymal transition and cancer stem cell features; 3) immune-associated TNBC; 4) luminal/apocrine TNBC with androgen-receptor overexpression; and 5) HER2-enriched TNBC. For each defined subtype, we highlight the major biological pathways and discuss potential targeted therapies in TNBC that might abrogate disease progression. However, many of these potential targets need clinical validation by clinical trials. We have yet to know how we can enrich the targets by molecular classifications.

## INTRODUCTION

Triple-negative breast cancer (TNBC), which accounts for 10–20% of all breast cancers, does not express estrogen receptors (ERs) or progesterone receptors (PRs) and lacks human epidermal growth factor receptor-2 (HER2) amplification. Patients diagnosed with TNBC have a higher risk of disease relapse within 5 years than patients treated for other breast cancer subtypes [1]. Thus, identification and evaluation of new biomarkers and therapeutic agents is a high priority. Because TNBC is a heterogeneous disease, many pathological and immunohistochemical subclassification have been proposed in the past decade to define more homogeneous subtypes. More recent advances have focused on disease stratification through the use of genome-wide approaches. Such “molecular portraits” of breast cancer are envisioned to provide a rationale for breast cancer prognosis and prediction to therapy. In this article, we sought to understand the classifications of TNBC based on similar gene-expression signatures and biological functions and their clinical relevancy.

### Search strategy and selection criteria

We conducted a Medline search up to December 2014 with use of the terms “triple negative” and “breast neoplasm” and/or search strings connected to the topics of interest—e.g., “classification”, “gene expression profiling”, “drug therapy”—without restrictions to date. Furthermore, references cited in the retrieved articles were screened for additional articles. Moreover, proceedings from the American Society of Clinical Oncology conference, the European Society of Medical Oncology conference, and the San Antonio Breast Cancer Symposium were researched in the abstract book for relevant presentations. We excluded publications not written in English, with impact factor < 3, with less than 10 citations (except for papers published less than 12 months ago). We also tried to select only one paper per team (except for gene expression profiling papers). Then, based on abstract concordance to our subject, we reviewed 315 papers. Ongoing clinical trials were searched using the http://clinicaltrials.gov site and referenced by their National Clinical Trial (NCT) number.

We initially screened for overlapping classifications of TNBC in whole-genome gene-expression profiling (GEP) papers and identified four majors classifications: the claudin-low [2], the intrinsic-PAM50 [3], and the molecular subsets described by Burstein *et al* [4]. and Lehmann *et al* [5]. We also searched for validated gene-expression signatures and markers of specific biological functions in TNBC populations. We cross-referenced the obtained information to identify clinically actionable TNBC groupings with similar gene-expression signatures, biological functions, and clinical outcomes. Although, recent papers highlighted the similarities and discrepancies of intrinsic PAM50 subtyping and Lehmann's seven subtypes [6–8], our incomplete knowledge of TNBC - validated gene signature, biomarkers or targeted therapies - precludes our ability to provide a consensus on clinically achievable TNBC subgrouping. Due to the lack of consensus on comprehensive treatment strategies for TNBC, we tried to re-organize the classification into theranostic subgroups with clinical relevance: detectable targets/pathway aberrations and available/potential targeted therapy.

### TNBC molecular subtypes with future clinical relevance and potential therapeutics

**Figure 1:**
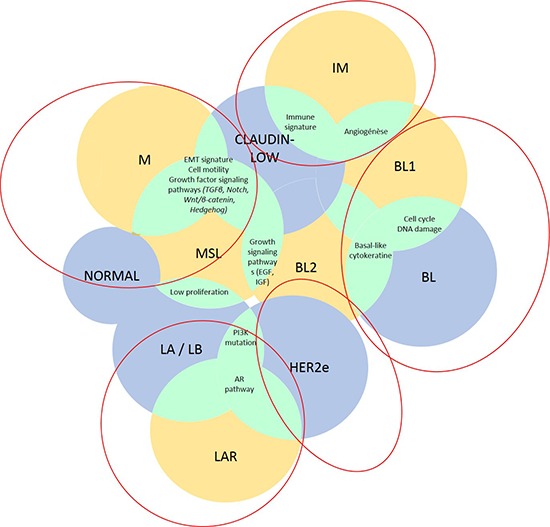
TNBC classifications Lehmann's *(yellow)*, PAM50/claudin-low *(blue)* classifications and their potent overlaps *(green)* are shown in this figure. **Abbreviations**: AR, androgen receptor; BL, basal-like; EGF, epidermal growth factor; HER2, human epidermal growth factor receptor 2; IGF, insulin growth factor; IM, immunomodulary; LAR, luminal androgen receptor; M, mesenchymal; MSL mesenchymal stem-like; TGFβ, transforming growth factor β.

We here provide five molecular groupings of TNBC that may have the greatest potential for clinical trial development using major previously published molecular classifications (PAM50 subtyping, claudin-low, Burstein's four subtypes and Lehmann's seven subtypes): 1) basal-like TNBC (BL-TNBC), characterized predominantly by DNA-repair deficiency but also growth factor pathway expression; 2) mesenchymal-like TNBC (ML-TNBC), with epithelial to mesenchymal transition (EMT) and cancer stem cell (CSC) features; 3) immune-associated TNBC (I-TNBC); 4) luminal/apocrine TNBC (LA-TNBC), with androgen receptor (AR) overexpression; and 5) HER2-enriched TNBC (HER2e-TNBC) (Figure 1). Next, we highlighted the key molecular pathways that are represented in these groups, with a specific interest towards identifying potential therapies that could be utilized to target each disease.

### Basal-like TNBC

The predominant molecular grouping of TNBC is BL-TNBC, which makes up approximately 25% to 80% of TNBC cases, depending on the definition used. Published definitions have been based on either immunohistochemical (IHC) characterization (CK5/6+; epidermal growth factor receptor (EGFR)+; ER-; HER2-) or GEP, without definitive consensus [9]. Although a matter of debate, several common characteristics have been observed in both descriptions of BL-TNBC, including high proliferative capacity and overexpression of BL cytokeratin genes (keratin-5 and -14) [5, 10].

According to Lehmann *et al*., BL-TNBC can be separated into two subgroups, basal-like 1 (BL1) and basal-like 2 (BL2) [5]. Profiling studies indicate that the BL1 subgroup is heavily enriched in the cell cycle-related genes and in DNA-damage repair pathways, which may be expected in highly proliferative tumors [5]. 25% of sporadic breast cancers harbor a deficiency in the DNA-repair, mainly in homologous recombination (HR) when double stranded DNA breakage occurs – similar to the genetic deficiency of *BRCA1* or *BRCA2* mutation carriers—leading to a subtype referred to as “BRCAness” [11, 12]. BL2 subgroup on the other hand is uniquely enriched in growth factor signaling pathways like EGF, MET pathway as well as IGF1R pathway.

BL-TNBC has one of the highest pathologic complete response (pCR) rates following chemotherapy [13]. As a group, targeting DNA-repair deficiency appears to be a promising treatment for BL-TNBC with BRCAness characteristics or BRCA-mutations (Table 1, Figure 2, and Supplementary Table 1). However, when one takes a close look, there was a significantly large difference in pCR rate between BL1 (51%) and BL2 (0%) subgroups, raising serious concerns about therapeutic applications whether to consider BL1 and BL2 as the same entity [8]. However, this requires prospective validation in large cohort of patients with TNBC. However, we can speculate that BL2 tumors display a gene signature that suggests activation of receptor tyrosine kinase pathways, suggesting that this subgroup may need to be grouped together with other subgroups harboring enriched growth factor/receptor tyrosine kinase pathways like mesenchymal like subgroup (see ML-TNBC) [5].

**Table 1:** Potential therapeutic approaches based on TNBC classification

Potential Therapeutic Subgroups	Drug class	Drugs (Given alone or with standard CHT)	Ongoing Trials (Phase)	Published Results of Clinical trials
Basal				
DNA-repair deficiency	Platinum	Cisplatin	NCT01672671(2) NCT01982448(2)#	50% of good pathologic response (Miller-Payne 3 to5) [14].
		Carboplatin	NCT01752686(3) NCT00532727(3)	48% of pCR when associated with standard CHT [15]. 68% and 33% of ORR for carboplatin and docetaxel for BRCA mutant patients [19].
	PARP inhibitors	Olaparib	NCT00707707(1)	37% of partial response when associated with weekly paclitaxel [99].
		Veliparib*	NCT02032277(3)	
Cell cycle	CDK inhibitors	Dinaciclib	NCT01624441(1)	
		P276–00*	NCT01333137(1)	
Mesenchymal				
Notch	GSI	RO4929097	NCT01151449(2)	
Hedgehog	SMO inhibitors	Erismodegib	NCT01757327(2)	
c-MET	c-MET-TKI	Tivantinib	NCT01542996(2)	Inactive as monotherapy [100].
	MET-mAb	Onartuzumab	NCT01186991(2)	No PFS improvement when added to CHT and bevacizumab [38].
Targeted therapies combination	GSI – SMO inhibitors	RO4929097 – Vismodegib	NCT01071564(1)	
Immune-associated				
Tumor vaccine	MUC-1 vaccine	MUC1 peptide vaccine	NCT00986609(0)	
Immune checkpoint blockade	Anti-PD-L1	Pembrolizumab MPDL3280A	NCT01848834(1) NCT01375842(1)	ORR of 16.1% in advanced TNBC [101]. ORR of 33% in metastatic TNBC [102].
CSF-1R	CSF-1R inhibitors	PLX3397	NCT01596751(2)	
Luminal/Apocrine				
AR	Androgen biosynthesis inhibitor	Abiraterone acetate	NCT01842321(2)#	
	AR inhibitor	Enzalutamide*	NCT01889238(2)#	42% CBR after 16 weeks [60]
		Bicalutamide	NCT02353988(2)# NCT02348281(2)#	
HDAC	HDAC inhibitor – Endocrine therapy	Entinostat – AnastrozoleLHB589 – Tamoxifen	NCT01234532(2) NCT01194908(2)	
Overlapping potential targets				
EGFR	EGFR mAb	Cetuximab*	NCT00463788(2)	ORR and PFS doubled with cetuximab and cisplatin [70].
		Panitumumab*	NCT00894504(2)	80% ORR when added to CHT [71].
	EGFR-TKI [69]	Gefitinib	NCT01732276(2)#	
		Erlotinib	NCT00491816(2)	
PI3K/AKT/mTOR	Pan PI3K inhibitor	Buparlisib	NCT01629615(2)	
			NCT01790932(2)	
		Pictilisib*	NCT01918306(2)#	
	mTOR inhibitor	Everolimus*	NCT01939418(2) NCT01931163(2)	
MAPK	MEK inhibitor	Trametinib	NCT01467310(B)	
VEGF	VEGF mAb	Bevacizumab*	NCT01898117(2)	Adjuvant setting: No improvement in DFS [85]. Metastatic setting: 35% reduced risk of relapse and a 19% RR without improved OS [86].
	VEGFR-TKI	Sorafenib*	NCT01194869(2)	
		Sunitinib*	NCT00887575(2)	Monotherapy showed no efficacy compared to SOC in previously treated advanced TNBC [103].
		Tivozanib	NCT01745367(2)	
		Apatinib	NCT01176669(2)	
	VEGFR and c-MET-TKI	Cabozantinib	NCT01738438(2)#	
Targeted therapy combinations	MEK inhibitor - AKT inhibitor	Trametinib - GSK2141795	NCT01964924(2)	
	VEGF inhibitor - c-MET inhibitor	Bevacizumab – Onartuzumab	NCT01186991(2)	

Based on our Classification, drugs specially investigated on a cohort of TNBC patients are listed in this table. These drugs are presented according to the pathway they target and thus illustrate our TNBC grouping.

* Ongoing trials evaluating targeted therapy in combination with platinum-based regimens.

# Trials enrolling TNBC patients.

**Figure 2:**
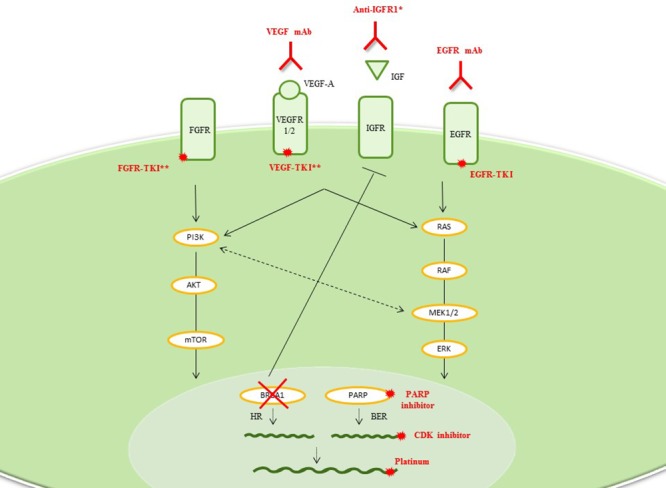
Basal-like TNBC Drug classes previously evaluated or currently being investigated in breast cancer clinical trials are shown. *More than 100 studies of anti-IGFR1 therapy (anti-receptor Abs, anti-ligand Abs, receptor-TKIs, and metformin) have been conducted. **Many combination drugs are currently being investigated: EGFR and HER2-TKIs (e.g., lapatinib and BIBW2992), c-MET and VEGFR-TKIs (e.g., cabozantinib) or FGFR and VEGFR-TKIs (e.g., lucitanib, dovitinib, BIBF1120). **Abbreviations**: BER, base-excision repair; CDK, cyclin-dependent kinase; EGFR, epidermal growth factor receptor; FGFR, fibroblast growth factor receptor; IGFR, insulin growth factor receptor; HR, homologous recombination; mAb, monoclonal antibody; TKI, tyrosine-kinase inhibitor; VEGFR, vascular endothelial growth factor receptor.

#### Platinum-based chemotherapy

Given the defective DNA-repair system as described above, the targeting DNA-repair deficiency appears to be a promising treatment for BL-TNBC with BRCAness characteristics or BRCA-mutations (more likely BL1: Table 1, Figure 2, and Supplementary Table 1). Chemotherapies based on the DNA cross-linking agent platinum could be effective for sporadic or germline DNA-repair-deficient breast cancers. Indeed, good response rates (RRs) to such agents have been associated with low BRCA1-mRNA expression and BRCA1 methylation [14, 15]. Platinum-based chemotherapy appears to significantly increase the pCR rate in TNBC patients [15, 16]. However, these results seem to be driven by patients with a family history of cancer and/or BRCA-positivity, who have a higher pCR rate (49%–64%) than the rest of the population (40%) [17]. In the metastatic setting, patients harboring BRCA mutations responded better to carboplatin than docetaxel. If BRCAness signature (as estimated by HR deficiency assay) appeared as a potent biomarkers of platinum sensitivity in TBCRC009 trial; it was not predictive of platinum response in the phase III TNT trial [18, 19]. These data support the use of platinum-based regimens for BRCA-mutant TNBC. As a result, carboplatin has been incorporated into combinatorial treatment in TNBC patients; however more careful selection of patients using BRCAness testing should be warranted.

#### Poly-ADP ribose polymerase (PARP) inhibitor

Alterations in DNA-damage response and repair mechanisms can lead to genomic instability and carcinogenesis, but may also offer a target for treatment in highly proliferative tumors such as BL-TNBC. Indeed, HR dysfunction has been shown to sensitize breast cancer cells to PARP inhibition, resulting in cell cycle arrest and apoptosis [20]. Preclinical data demonstrated that PARP inhibitor olaparib has antitumor activity in BRCA-mutant cell lines [21], this was later confirmed in a phase II trial [22]. The phase III study of iniparib did not meet this goal, with the negative result attested to iniparib's poor inhibitory action against PARP [23, 24]. New, highly potent PARP inhibitors, such as BMN-673 are in early stage development and the results of their clinical utility eagerly awaited [25]. Combination treatment appear promising based on preliminary results of the I-SPY2 trial which reported a doubled pCR rate when veliparib and carboplatin were added to standard neoadjuvant chemotherapy regimens [26]. Another interesting observation is that CDK inhibitors provided a therapeutic target for PARP inhibitors in BRCA-competent cell lines by increasing DNA damage [27]. Therefore, smart combination strategy may create a novel susceptibility to DNA repair targeting therapeutics in non BL1 TNBC, and can be used as one strategy to induce pathologic response to the chemotherapy.

### Mesenchymal-like TNBC

Mesenchymal, mesenchymal stem like, claudin-low are generally indicating the subgroups of TNBC that harbor mesenchymal like features—represented by enriched genes involved in EMT and the biological regulation of CSCs [2, 5]. Interestingly, most mesenchymal stem-like (MSL) samples are usually classified as normal-like, whereas most mesenchymal (M) tumors are classified as Basal-like when tested by PAM50 (when the “claudin-low” subgroup is not considered) [7]. It could actually suggest that the MSL group of tumors is actually composed of tumors highly contaminated by normal breast tissue. These discrepancies were also noticed in the smaller cohort of Burstein *et al*.: MSL overlapped with a mesenchymal subgroup whereas M tumors were mostly Basal-Like Immune-suppressed (BLIS) [4].

**Figure 3:**
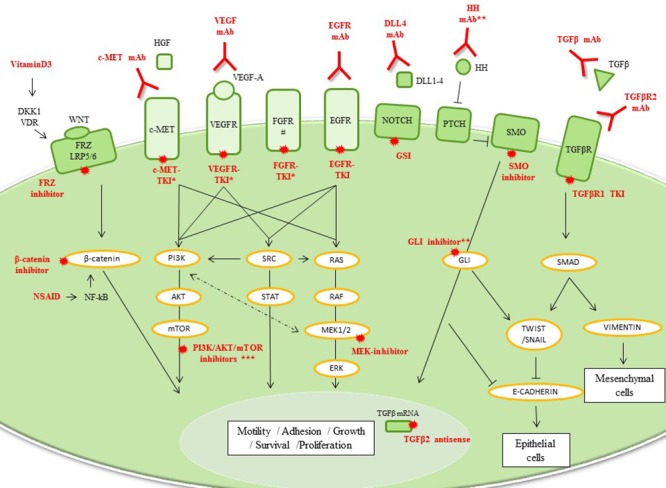
Mesenchymal TNBC Drug classes previously evaluated or currently being investigated in breast cancer clinical trials are shown. *Many combination drugs are currently being investigated: EGFR and HER2-TKIs; c-MET and VEGFR-TKIs or FGFR and VEGFR-TKIs. **Not yet in clinical trials. ***PI3K/AKT/mTOR inhibitors are detailed in luminal/apocrine-TNBC part (Figure 4). # PDGFR and IGFR are also overexpressed in ML-TNBC and are targetable. However, current therapies targeting PDGFR have a large overlap with those that target VEGF. Imatinib, an anti-PDGF agent, had potential immunosuppressive effects and no antitumor activity in metastatic breast cancer overexpressing PDGFβ. **Abbreviations**: DKK1, Dickkopf-homolog-1; DLL, delta-like ligand; EGFR, epidermal growth factor receptor; FGFR, fibroblast growth factor receptor; FRZ, Frizzle receptor; GSI, γ-secretase inhibitor; HGF, hepatocyte growth factor; HH, Hedgehog ligand; mAb, monoclonal antibody; NF-κB, nuclear factor κ-beta; NSAID, non-steroidal anti-inflammatory drug; PTCH, patched receptor; SMO, smoothened; TGFβ, transforming growth factor β; TKI, tyrosine-kinase inhibitor; VDR, vitamin D receptor; VEGFR, vascular endothelial growth factor receptor.

During EMT, epithelial breast cancer cells acquire the expression of mesenchymal markers while losing the expression of epithelial-related genes involved in the maintenance of cellular junctions. While comparing EMT markers, it was recognized that elevated vimentin and decreased E-cadherin protein expression in TNBC cells could stratify a mesenchymal-TNBC subgroup (Supplementary Figure 1). From the signaling pathway perspective, the activation of EGFR, which is frequently overexpressed in TNBC, has been implicated in EMT, as have other tyrosine-kinase receptors (e.g., c-MET, fibroblast growth factor, insulin growth factor [IGF], platelet-derived growth factor) [28, 29]. Other pathways, including the transforming growth factor β (TGFβ), Notch, and Wnt/β-catenin signaling pathways [29], are also involved in EMT, and many of these are heavily enriched in ML-subtype [5]. Mesenchymal cells also harbor CSC-like features, the hallmarks of metastatic potential. In an unsupervised analysis of a collection of breast cancer cell lines, Neve *et al*. described a cluster of TNBC cells that exhibited a CSC-like expression profile [30]. Moreover, the induction of a CSC profile or the expression of mesenchymal markers in breast cancer cells have been correlated with chemotherapy resistance [31]. CSC-like features, consistent with EMT phenotype, are known to be driven by many well-known signaling pathways such as the MAPK and Wnt pathways, which suggest that inhibitors of these pathways should be utilized in conjunction with standard chemotherapy. Many promising EMT-targeted and CSC-targeted treatments are under investigation in early stage clinical trials (Table 1, Figure 3 and Supplementary Table 1).

#### CSC regulators

Activation of Wnt/β-catenin signaling correlates with the expression of CD44+/CD24- [32]. Despite the inherent difficulties in developing novel Wnt inhibitors, many drugs already approved by the U.S. Food and Drug Administration inhibit Wnt pathways, including vitamin D3, non-steroidal anti-inflammatory drugs and some antibiotics (e.g., salinomycin) [33]. The Notch signaling pathway also has a crucial role in maintaining breast CSCs, and thus may provide a therapeutic avenue in ML-TNBC. To date, two kinds of Notch inhibitors have been developed for oncological purposes: γ-secretase inhibitors (GSIs) and delta-like ligand 4 monoclonal antibodies (mAbs). In preclinical studies, GSIs have been found to sensitize chemoresistant cell populations, including CSC-like tumor cells [34]. Importantly, functional study suggested that PEST domain mutations of Notch receptors are frequent in TNBC and active Notch pathway conferring GSI sensitivity [35]. Given that Hedgehog ligand, GLI expression and SMO are also up regulated in TNBC, there is a potential opportunity to therapeutically target this pathway; however, no clinical trials have been designed specifically for ML-TNBC.

#### c-MET targeted therapy

Targeting this pathway in ML-TNBC could also be successful since c-MET signaling can control EMT and CSC phenotypes [29]. Moreover, stromal secretion of hepatocyte growth factor was recently demonstrated to activate the c-MET pathway and leads to resistance to EGFR-TKIs in breast cancers, an effect already well-known in non-small cell lung cancer [36]. Appropriately, co-inhibition of EGFR and c-MET suppressed tumor growth in preclinical models [37]. However, initial results with c-MET inhibitors were disappointing, with no improvement of progression-free survival when onartuzumab was given with paclitaxel/bevacizumab-based regimens in unselected metastatic TNBC patients [38]. However, with better understanding of pathway and possible expanded target group including BL2 TNBCs showing enriched in genes associated with the c-MET pathway [5], this pathway still holds future potential.

#### TGFβ inhibitors

In a preclinical trial, TGFβ tyrosine-kinase inhibitor (TKI) induced a tremendous mesenchymal-to-epithelial transition (MET) reversing EMT in CD44+ breast cancer cells, which justified its further development [39]. Initial results from studies of trabedersen, an antisense oligodeoxynucleotide directed against TGFβ2 mRNA, revealed promising efficacy in solid tumors known to overexpress TGFβ2 ligand [40].

### Immune modulatory/associated TNBC

The “immunomodulatory” subtype is enriched in gene ontologies of the immune cell process including immune cell signaling (B, T, and NK cells), cytokine signaling, antigen processing-presentation, and core immune signal transduction pathways [5]. Compared with the other intrinsic groups defined by Sorlie *et al*. [3], claudin-low tumors expressed a high level of immune system response genes (B-cell, T-cell, and CD8-signatures) [2]. Additionally, Burstein *et al*., using an RNA-based gene profiling, identified a good prognostic subset called “Basal-like immune activated” (BLIA), overexpressing CTLA-4 on top of other genes overexpressed in immunomodulatory TNBC [4].

While different groups proposed slightly different analysis to define the group, it is clear that there are subset of TNBC that harbor a lot of modulatory signatures dictated by immune systems. The immune response signature was correlated with enhanced levels of immune cell infiltration and resulted in good clinical outcome in TNBC [2, 41–44]. Tumor-infiltrating lymphocytes seem predictive of neoadjuvant chemotherapy response [45, 46].

**Figure 4:**
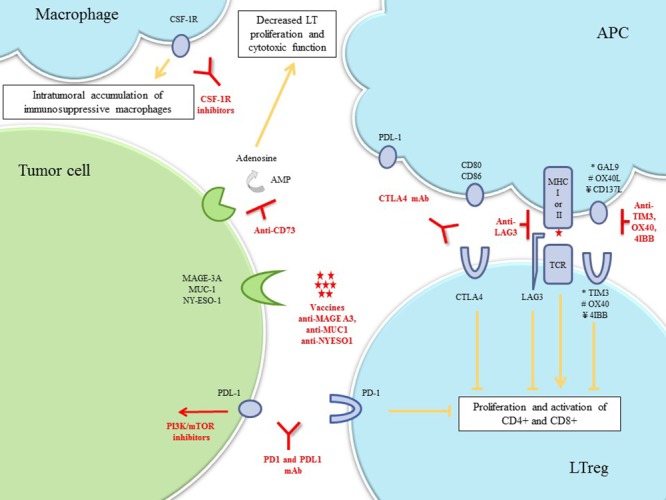
Immune-associated TNBC Drug classes previously evaluated or currently being investigated in breast cancer clinical trials are shown. **Abbreviations**: AMP, adenosine monophosphate; APC, antigen-presenting cell; CSF-1R, colony stimulating factor-1; CTLA-4, cytotoxic-T-lymphocyte-antigen-4; LT lymphocyte T; LTreg, lymphocyte T regulator; mAb, monoclonal antibody; MAGE-A3, melanoma-associated-antige3; MHC, major histocompatibility complex; MUC1, mucin-1; PD-1, program-death-1; PDL-1, program-death-ligand-1; TCR, T-cell receptor.

Immune-based therapies are actively developed in breast cancer (Table 1, Figure 4, and Supplementary Table 1). Whether specific immune system response gene amplification and/or immune pathway enrichment is predictive of treatment efficiency remains to be known. Due to lack of evidence so far, tumors with one of the characteristic were gathered in a common immune subgroup.

#### Immune checkpoint blockade

Immune checkpoint pathways are an elaborate series of cellular interactions that prevent the excessive activity of T-cells under normal conditions. In immune-associated TNBC, inhibiting these checkpoints and enhancing T-cell activity against tumor cells could be used therapeutically. Activation of cytotoxic-T-lymphocyte-antigen-4 (CTLA-4), a cell surface receptor of lymphocyte T regulators, down-modulates the amplitude of T-cell activation. Two anti-CTLA-4- mAbs, ipilimumab and tremelimumab, are currently being evaluated in breast cancer, but no trial currently exists to assess their efficacy in the various stratifications of TNBC. Programmed-cell-death-1 (PD-1) and its ligand, PD-L1, are overexpressed in 20% of TNBCs [47]. The PD-L1/PD-1 pathway is a potent mechanism by which immunogenic tumors evade host immune response. Anti-PD-1 and anti-PD-L1-mAbs disrupt this ligand-receptor interaction, thereby enhancing T-cell immune response. PD-L1 expression appears to be a potential predictive biomarker of objective response rate (ORR) [48]. Inhibiting PTEN up-regulates PD-L1 expression, suggesting that agents targeting the PI3K pathway might be effective to enhance the antitumor adaptive immune response to TNBC [47]. The combination of cell signaling pathway inhibitors plus immune checkpoint blockade drugs needs to be explored in TNBC (Table 1 and Figure 4).

#### Tumor vaccines

Breast cancer vaccines targeting tumor antigens have been investigated for the past decade. Although no TNBC-specific antigen has been validated, several targets in breast cancer cells have been identified, including NY-ESO-1, MAGE-A3, and MUC-1 [49, 50]. NY-ESO-1 expression has been identified in 12–24% of TNBC patients, among whom 73% had high Ab response to NY-ESO-1, indicating high immunogenicity [51, 52]. Interestingly, immune gene signature was predictive of MAGE-A3 specific immunotherapeutic response [53].

### Luminal/apocrine TNBC

LA-TNBC, despite lacking ERs and PRs, is enriched in hormonally regulated pathways. Indeed, AR overexpression can replace ER expression as a major component of steroid-related signaling [5, 10, 54]. This subgroup, which could include the LAR, the luminal A-B, the Burstein's LAR and molecular apocrine subtypes, shares other features including high luminal gene expression, lack of basal-cytokeratin markers, and low proliferation rate [5, 10, 54]. AR-positivity, defined as nuclear staining in at least 10% of cancer cells, has been detected in approximately one-third of TNBCs and is associated with good prognosis [55–57]. Interestingly, a low pCR rate of 6%-10% was observed in the luminal/apocrine setting, after preoperative chemotherapy but there was trend for a better prognosis [8, 56].

#### AR inhibitors

Sensitivity to bicalutamide, an oral AR inhibitor, was better in LAR cell lines than in other subtypes and a recent phase II study confirmed the interest of such drug showing a 19% of clinical benefit rate at 24 weeks for ER/PR-negative AR-positive breast cancer patients [5, 58]. Enzalutamide, a new generation anti-androgen, abrogated AR-mediated proliferation *in vitro*, and yielded 42% of clinical benefit rate at 16 weeks in advanced AR-positive TNBC [59, 60]. Another compound, enobosarm, yielded a 35% clinical benefit in metastatic AR-positive breast cancer [61]. Recent data suggested that even non-LAR subtypes with relatively lower AR expression may also benefit from AR targeted therapy [62]. Various efforts to streamline the testing of AR in breast cancer and develop effective AR targeting treatment are currently made by researchers.

#### Histone deacetylase (HDAC) inhibitors

**Figure 5:**
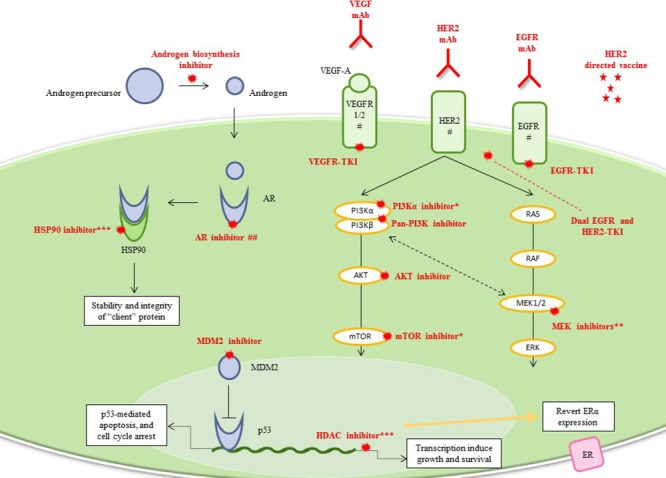
Luminal/apocrine TNBC and HER2-enriched TNBC Drug classes previously evaluated or currently being investigated in breast cancer clinical trials are shown. *Dual PI3K/AKT/mTOR inhibitors are on the way. **Current mechanistic-driven combination drugs use MEK inhibitors with: AR inhibitors, PI3K inhibitors, AKT inhibitors, and dual PI3K/mTOR inhibitors. ***Inhibition of HSP90, a molecular chaperone essential for the stability and integrity of various “client” proteins such as AR, is an attractive therapeutic target for cancer. # Anti-HER2, anti-EGFR, and anti-VEGF treatments are more specific for HER2-enriched TNBC. ## AR signaling has an important role in the molecular apocrine subgroup, which mainly overlaps the luminal-TNBC subgroup but could also overlap the HER2e-TNBC subgroup because of the high frequency of HER2 amplification. **Abbreviations**: AR, androgen receptor; EGFR, epidermal growth factor receptor; HER2, human epidermal growth factor receptor 2; HDAC, histone deacetylase; HSP90, heat-shock protein 90; mAb, monoclonal antibody; MDM2, mouse double minute 2; TKI, tyrosine-kinase inhibitor; VEGFA, vascular endothelial growth factor A; VEGFR, vascular endothelial growth factor receptor.

As HDAC regulates AR target genes in prostate cancer cells, HDAC-inhibitors were tested in TNBC and had low toxicity [63]. Furthermore, in-vivo data suggest that HDAC inhibitors cause cells with the TNBC phenotype to express ER and become sensitive to endocrine therapy [64]. Thus, clinical trials of HDAC-inhibitors and aromatase inhibitors in TNBC patients are underway (Table 1, Figure 5 and Supplementary Table 1).

### HER2-enriched TNBC

Six to eight percent of TNBCs are considered to be HER2e [3]. In Lehmann's molecular classification of TNBC, HER2 did not appear as an independent classifier, but instead the majority of HER2e tumors segregated into LAR and BL2 subgroups [5, 10]. Indeed, HER2e-TNBC shared characteristics with LA-TNBC including PI3KCA mutations and high levels of luminal-like genes such as AR [54, 65]. Therefore, while we suggest this subgroup as a separate clinical entity, it is possible that LA-TNBC and HER2-enriched group can be grouped together in near future based on further scientific discoveries. Although the clinical role of minimal HER2 expression (HER2 1+, 2+ by IHC) in TNBC is largely unknown, there are opportunities to evaluate HER2-targeted therapies (Figure 5 and Supplementary Table 1).

#### HER2-targeted therapy

Central IHC analysis of breast cancer samples from the NSABP-B31 trial revealed that 10% of patients receiving adjuvant trastuzumab were actually HER2-negative. In this population, trastuzumab had a clinical benefit ; however, the results of this subset analyses should be taken with extreme caution [66]. The phase III NASBP B47 trial should determine the clinical relevance of HER2-targeted therapy in HER2-low expression (IHC 1+ or 2+) TNBC.

#### HER2-directed vaccine

Two recent phase II studies demonstrated a benefit of the HER2 peptide vaccine AE37, in patients with HER2-low expression, especially those with TNBC (60% relative reduction in recurrence). Phase III in TNBC are warranted [67, 68].

### Overlapping pathways and potential therapeutics

In our literature review, we noticed several signaling pathways that were common to multiple groups of TNBC. While this was not unexpected, it does suggest that investigations into therapeutics that inhibit these pathways should provide serious consideration to which subgroups of TNBC will be tested.

#### EGFR targeted therapy

EGFR is overexpressed primarily in ML-, BL-, and HER2e-TNBC [5, 10, 65]. EGFR and its downstream signaling pathways consequently appear as promising targets in these TNBC subsets [69]. In a xenograft model of ML-TNBC, treatment with an EGFR-TKI induced MET transition and subsequent tumor regression [28]. Various EGFR-TKIs given as monotherapy did not provide any clinical benefit in a cohort of unselected patients [69]. More concordant with preclinical results, EGFR mAbs combined with chemotherapy showed promising ORRs in TNBC patients [70, 71]. Complementary data showed minor inactivation of EGFR pathway in TNBC by EGFR inhibitors, potent testimony of activation of additional resistant pathways [72].

#### Fibroblast growth factor receptor (FGFR) targeted therapy

Because FGF2 ligand is highly express in BL-[73], and FGFR is highly expressed in ML- and BRCA-associated tumors [5, 74], two kinds of FGFR inhibitors are being investigated in breast cancer: TKIs targeting both VEGFRs and FGFRs and pan-FGFR TKIs. In the first instance, results from a phase II trial of dovitinib in FGFR1-amplified breast cancer suggest that anti-FGFR therapy results in stable disease in TNBC [75]. In the second instance; since genomic alterations of FGFR are predictive of BCJ398 sensitivity [76], current clinical trials have restrictive inclusion criteria for biological markers (FGFR1 and/or FGFR2 amplification, or FGFR3 mutation) but are open to various solid cancers. This approach is necessary to obtain a sufficient number of patients and significant results.

#### IGFR-targeted therapy

IGFR-related signaling genes are heavily enriched in ML- and BL-TNBC. Excitingly, targeting this pathway in BL-TNBC may be highly effective as BRCA-deficient cells, unlike wild-type cells, cannot down-regulate IGFR expression [77]. However, neither BRCA status nor IGFR-1 plasma levels are biomarkers for anti-IGFR treatment. The biomarker issue raised for anti-IGFR therapy is common in oncology and also applies to studies investigating the VEGF pathway. Thus, phase III results in unselected patients have been disappointing [78].

#### PI3K/AKT/mTOR pathway targeted therapy

Up to 45%, 39%, and 29% of “intrinsic” luminal-A, HER2e, and luminal-B, respectively, have PI3KCA mutations [65]. Preliminary data suggest that these mutations increase the sensitivity of cancer cells to PI3K/AKT/mTOR inhibitors [79]. More controversial is the impact of PTEN loss as a predictor of treatment efficacy [5, 79]. Phase I trials showed that class I pan-PI3K inhibitors elicited disease stabilization or partial response in TNBC [80]. At the same time, a beta-sparing PI3K inhibitor showed promising preliminary clinical activity in PI3KCA-mutant breast cancers [81]. Dual PI3K and mTOR inhibitor NVP-BEZ235 had a potent effect on ML- and LA-TNBC cell lines [5]. This sensitivity was confirmed in a phase I study [82]. In the future, laboratory marker analysis should be performed to determine the precise roles of PI3K mutation and PTEN loss and better select patients who would benefit from these targeted treatments.

#### MAPK pathway targeted therapy

MEK inhibitors appear to be a promising agents in ML-TNBC because the ERK1/2 pathway is overexpressed in this subtype [5]. If only 2% and 5% of all breast cancers have BRAF and KRAS mutations, respectively, a “RAS-like” transcriptional program confers sensitivity to MEK inhibitors in preclinical models of BL-TNBC [83]. In contrast, MEK inhibitors should not be relevant in LA-TNBC as PTEN loss is a negative predictor of MEK-inhibitors' efficiency [83]. An obvious feedback loop between the PI3K/AKT/mTOR and RAS/RAF/MEK/ERK pathways has direct clinical implications, as MEK inhibition leads to PI3K activation and vice versa [83]. Moreover, inhibition of MAPK activity restore ER expression and endocrine therapy response *in vitro* [84]. Even if single-agent MEK inhibitor may not be the most relevant treatment for TNBC, combination therapy using this drug may have clinical efficacy in TNBC.

#### Angiogenesis inhibitors

Three kinds of anti-angiogenic agents are currently on the market: anti-VEGF-A mAbs (e.g., bevacizumab), pan-VEGFR TKI (e.g., sunitinib, sorafenib, pazopanib), and VEGF-trap (e.g., aflibercept). The U.S. Food and Drug Administration's approval of bevacizumab for breast cancer was withdrawn because of insufficient benefit and consequent adverse effects in the global breast cancer population. In the adjuvant setting, adding bevacizumab to chemotherapy did not improve disease-free survival in unselected TNBC patients [85]. A meta-analysis of three phase III trials suggested that bevacizumab reduces the risk of progression of metastatic TNBC by 35% [86]. Interestingly, claudin-low, basal, and HER2e display a VEGF-signature, sign of angiogenesis [87]. Concordant data suggest higher intratumoral expression of VEGFA in BL-TNBC and HER2e-TNBC, than in the ML-TNBC [88, 89]. There are no predictive biomarkers for angiogenesis inhibitors, yet. To obtain any relevant results and improve outcomes with the use of anti-angiogenic agents, we will need to preselect patients using predictive biomarkers.

#### TP53 mutation targeting

p53 mutations are detected in 100%, 85% and 40% of HER2e-, basal and luminal TNBCs, respectively [10]. Targeting the p53 pathway can be both direct and indirect. E.g., MDM2 inhibitors could reactivate p53's tumor suppressor function in non-mutant tumors [90]. Defective P53-mediated cell-repair lead to G2-M dependency of cells, therefore offering a therapeutic strategy to target apoptosis in TNBC. Many apoptosis targeting agents are currently tested in solid tumor, and are recognized as upcoming drugs in TNBC.

### Therapeutic strategy and biomarker development

There are numerous targets among subtypes as we reviewed here in TNBC; however their activity as single agents in TNBC has proven or might be limited. Further, there is limited number of actionable single gene mutation drivers in TNBC. Therefore, biologically driven combinatorial therapies should be considered. An extensive number of active clinical trials are investigating combinatory-targeted treatments on the basis of agents' synergistic effect in preclinical studies [91]. Specifically, these combination therapies should be considered in TNBC, based on the compensatory pathways activated by single pathway inhibition by targeted therapy or preclinical data suggesting their synergy: such as, EGFR- and MEK- inhibitors; MEK-, MET- or PARP-inhibitors; VEGF- and mTOR inhibitors [92]. Further, because of the high rate of PI3K mutations in AR-positive tumors, PI3K- and AR- inhibitors should be pursued [93]. Although, multiple combination therapies are available to pursue as clinical trials, our incomplete knowledge of TNBC precludes our ability to provide a rationale for treatment prioritization.

Few clinical trials have assessed combination therapies in TNBC alone, and even fewer have assessed such therapy in the various TNBC subtypes (Table 1 and Supplementary Table 1). Thus, most trials are phase I studies involving various solid tumor types or unspecified breast cancer subtypes. Therefore, there is an urgent need to design clinical trials that assess drug efficacy in TNBC specifically, as this effect may be unobserved in studies involving a large unspecified population. We speculate that some drugs, such as EGFR-TKIs or VEGF-mAbs, may have already suffered from designs with incomplete annotation.

Additionally, a large proportion of preclinical studies utilize only a handful of established human cell lines that commonly do not represent all TNBC subtypes. For example, the TNBC cell line, MDA-MB-231, has been the workhorse of preclinical investigation for more than three decades and has provided much insight into the biology of primary tumor and metastases. However, MDA-MB-231 is a ML-TNBC cell line, and results derived from its study should be interpreted in the context of the ML-TNBC subgrouping. Preclinical studies assessing drug activity in TNBC should use multiple cell lines that encompass all molecular subtypes of the disease.

Another important element to consider is tumor evolution. Most tumor characterization is based on pretreatment core biopsies. However, tumor's genetic instability can cause changes in molecular characteristics at various times points of disease, as was demonstrated for PI3K pathway mutations [94]. So, despite the classification proposed, we do not know whether baseline classification will predict response after one treatment or even after weeks of natural tumor evolution. Furthermore, we do not know how these classifications will affect combination targeted therapy or combination treatments with conventional chemotherapy. Finally, we need to think about performing repeat core biopsies and developing less invasive measures of clinical assessment, such as monitoring circulating tumor cells and circulating free tumor DNA [95], to provide accurate and personalized treatment along the course of TNBC. Most clinical trials of targeted treatment enrolled heavily pretreated breast cancer patients without new characterization, but whether this is relevant is unknown. There is an urgent need to answer these questions to correctly design future studies.

Lastly, biomarkers for selecting patients for treatment are urgently needed [95]. A greater understanding of TNBC biology will uncover potential biomarkers that will facilitate clinical trials of novel treatments and the development of predictive biomarkers for these treatments. However, the main issue of how to identify biomarkers with high clinical validity and utility remains. PI3K mutation, VEGFR2/VEGF-A, BRCA1/2 mutations and ‘BRCAness’ have all been used to stratify patients in studies of anti-angiogenic agents or PARP inhibitor. With exception to BRCA1/2 mutations, no candidate biomarkers have been proven to have sufficient pragmatic validity in TNBC.

## CONCLUSIONS

Developing personalized therapies for TNBC requires a comprehensive understanding of the molecular basis of its oncogenic pathways and microenvironmental changes, as well as the effects of the immune system and therapies on these pathways. The more we understand the biology, the more we are prompt to split TNBC disease into multiple subgroups. However, multiplication of subtypes could yield to “orphan” TNBC disease and unnecessary splitting with major issue to design powerful trial with sufficient number of patients. To overcome this issue, our review suggests five possible major clusters of TNBC based on current knowledge and clinical trial development. Each of them harbors a dominant biological function/pathway, which could justify the above distinction. Our review identified four predominant function/pathway: DNA-repair deficiency, EMT and CSC, immune-associated, androgen-receptor overexpression. Although, multiple papers explored TNBC gene expression profiling, our incomplete knowledge of TNBC biomarkers precludes our ability to provide clinically achievable TNBC grouping with rationale for targeted therapies. Thus, in this review, we detailed for each pathway, various molecular-based treatments which are currently being investigated.

Thus, the first clinical need is to develop robust biomarkers that reflect the molecular behavior of TNBC, to generate more homogenous TNBC subgroups. To discover targetable route, we should also think in term of activated pathways and not restrict our research to mutational or expression data [96].

The second is to determine whether these molecular targets are clinically relevant to the treatments. Currently, testing new drugs without any correlated biomarkers studies might be a waste of time. Current insight of BRCA1/2 mutation and AR expression level in management of TNBC illustrate well the discussion.

The third is to prevent resistance to the proposed treatment. Many cancer centers are attempting to create comprehensive treatment strategies for TNBC so that personalization of treatment can be initiated.

**Figure 6:**
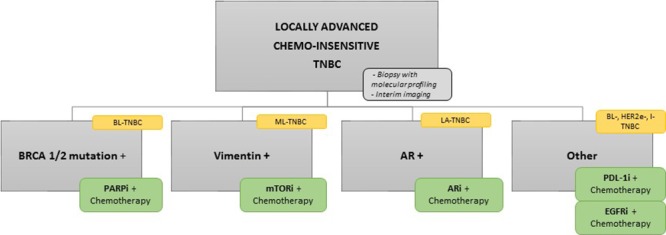
Flow chart of potent new TNBC clinical trial Potent new design on TNBC clinical trial based on the classification we detailed and the new targeted drugs currently in development in TNBC. **Abbreviations**: AR, androgen receptor; ARi, androgen receptor inhibitor; BL, basal-like; EGFR, epidermal growth factor receptor; EGFRi, epidermal growth factor receptor inhibitor; I, immunomodulary; LA, luminal/apocrine; ML, mesenchymal-like; mTORi, mTOR inhibitor; PDL-1i, PDL-1 inhibitor.

Based on our review, one approach could be first to select chemo-resistant population which can benefit from additional treatment using baseline molecular profiling and imaging during standard chemotherapy treatment [97, 98]. Secondly, patients could be selected based on their HR deficiency status and AR expression level. Remaining patients could be segregated into mesenchymal-TNBC or non-mesenchymal-TNBC to benefit from investigational treatments (Figure 6). Regardless of how you design your personalized treatment in TNBC, multiple specimen correction is needed in the course of clinical trials or providing standard of care because we have yet to discover a robust treatment outcome predictable biomarkers. For sure, we see the future of personalized therapy development in TNBC as based on biology-oriented comprehensive approaches. However, we do not know yet if the biological classification based on gene expression profile or gene mutation/amplification can truly enrich the targets in TNBC.

## SUPPLEMENTARY DATA FIGURE AND TABLE



## ABBREVIATIONS

AR: androgen receptor
BC: breast cancer
CDK: cyclin-dependent kinase
CHT: chemotherapy
DFS: disease-free survival
DR: death receptor
EGFR: epidermal growth factor receptor
GSI: γ-secretase inhibitor
HDAC: histone deacetylase
IAP: inhibitor of apoptosis protein
mAb: monoclonal antibody
MUC1: mucin-1
ORR: objective response rate
OS: overall survival
pCR: pathological response rate
PFS: progression-free survival
RR: response rate
SMO: smoothened
SOC: standard of care
TKI: tyrosine-kinase inhibitor
VEGF: vascular endothelial growth factor receptor
