# Supplementary material for: Is the future of personalized therapy in triple-negative breast cancer based on molecular subtype?
Source: Oncotarget. 2015 May 7;6(15):12890–908. doi: 10.18632/oncotarget.3849 (PMC4536987; doi:10.18632/oncotarget.3849)
Supplement: Supplementary file 1 [file oncotarget-06-12890-s001.pdf]

SUPPLEMENTARY DATA

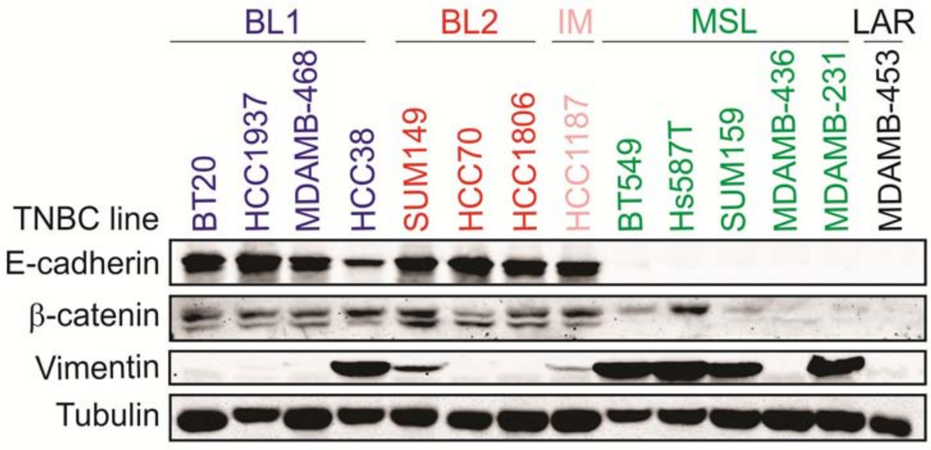

Supplementary Figure S1: Immunoblotting analysis of protein markers of epithelial-to-mesenchymal transition in a panel of TNBC cell lines. Gain of vimentin, and loss of E-cadherin expression appears to be a relevant biomarker for mesenchymal-TNBC.

Supplementary Table S1. Selection of ongoing trials and already published results for BCs

Potential Therapeutic Subgroups	Drug class	Drugs (Given alone or with standard CHT)	Ongoing trials (Phase)	Published results of Clinical trials
Basal				
DNA-repair deficiency	PARP inhibitors	Olaparib	NCT00494234(2) NCT01078662(2)	41% of partial and complete response.* [22] 31% of tumor response rate.* [1]
		Niraparib	NCT01905592(3)*	
		BMN673	NCT02034916(2)*	
		Veliparib	NCT01042379(2)	52% of pCR when associated with standard CHT and carboplatin. [26]
		Olaparib†	NCT01445418(1)	
Mesenchymal				
Wnt/β-catenin	Wnt inhibitor	LGK974	NCT01351103(1)	
	FRZ inhibitor	Vantictumab	NCT01973309(1)	
	β-catenin	PRI-724	NCT01302405(1)	
Notch	DDL4 mAb	MEDI0639	NCT01577745(1)	
Hedgehog	Hedgehog inhibitor	PF-04449913	NCT01286467(1)	
	Itraconazole		NCT00798135(0)	
TGFβ	TGFβ mAb	Fresolimumab	NCT01401062(1)	
	TGFβR mAb	IMC-TR1	NCT01646203(1)	
Immune-associated				
Immune checkpoint	Anti-CTLA-4	Tremelimumab		Increased number of LT effectors after treatment.** [2]
blockade		Ipilimumab	NCT00083278(2)	Activation of circulating LT after-treatment. [3]
	Anti-PD-L1	MPDL3280A	NCT01375842(1)	
	Anti-PD-1 - Anti-CTLA-4	Nivolumab-Ipilimumab	NCT01928394(2)	
Tumor vaccine	NY-ESO-1 vaccine	IMF-001	NCT01234012(1)	
	MUC-1 vaccine	MUC1 peptide vaccine		STn-KLH vaccine showed no benefit in time to progression or OS. [4]
Luminal/Apocrine				
AR	Androgen biosynthesis inhibitor	Abiraterone acetate	NCT00755885(2)#	
	AR inhibitor	Bicalutamide	NCT00468715(2)	19% of clinical benefit rate at 24weeks without objective response.# [58]

(*Continued*)

Potential Therapeutic Subgroups	Drug class	Drugs (Given alone or with standard CHT)	Ongoing trials (Phase)	Published results of Clinical trials
	SARM	Enobosarm	NCT01616758(2)	35% of clinical benefit.# [61]
HSP	Anti-HSP90	Tanespimycin	NCT00096109(2)	Stable disease with high toxicity profile. [5]
		Ganetespib	NCT01677455(2)	
HER2-enriched				
HER2	HER2-targeted therapy	Trastuzumab	NCT01275677(3)	Significant clinical benefit (RR 0.34).## [66]
		Pertuzumab		8% of stable disease greater than 6 months.## [6]
		Margetuximab	NCT01828021(2)	
	HER2-directed vaccine	E75-peptide vaccine	NCT01479244(3)	Better PFS with greater benefit for HER2-low (IHC 1+ or 2+) pts.## [7]
		AE37-peptide vaccine	NCT00524277(2)	60% of relative reduction risk for TNBC.## [67]
Overlapping potential targets				
EGFR	EGFR-TKI [69]	Gefitinib		No clinical benefit as monotherapy**
		Erlotinib		No clinical benefit as monotherapy**
FGFR	FGFR-TKI	BCJ398	NCT01004224(1)	
PI3K/AKT/mTOR	PI3Kα inhibitor	GDC-0032	NCT01862081(1)	
	AKT inhibitor	MK2206	NCT01319539(2) NCT01277757(2)	
VEGF	VEGF mAb	Bevacizumab	NCT01663727(3)	
	VEGFR-TKI	Sorafenib	NCT01234337(2)	PFS improvement of 2.3 months when associated to CHT.## [8]
		Pazopanib	NCT01498458(1)	20% of stable disease more than 6 months.[9]
		Sunitinib	NCT00393939(3)	ORR improved by 13% when associated with CHT. ## [10]
	VEGFR and c-MET-TKI	Cabozantinib		14% ORR.** [11]
	VEGFR and FGFR-TKI	Dovitinib	NCT01262027(2)	25% of response or stable disease in FGFR1-amplified/ER+ BC.## [12]

(*Continued*)

Potential Therapeutic Subgroups	Drug class	Drugs (Given alone or with standard CHT)	Ongoing trials (Phase)	Published results of Clinical trials
		Lucitanib	NCT01283945(2)	
			NCT02053636(2)	
		Nintedanib	NCT01484080(2)	
Combination of targeted therapies	PI3K inhibitor - PARP inhibitor	Buparlisib - Olaparib	NCT01623349(1)	
	PI3K inhibitor - SMO inhibitor	Buparlisib - Erismodegib	NCT01576666(1)	
	PI3K inhibitor- FGFR-TKI	BYL719 - BCJ398	NCT01928459(1)	
	PI3K inhibitor- MEK inhibitor	Buparlisib - MEK 162	NCT01363232(1)	
	PI3K-AKT inhibitor – MEK inhibitor	SAR245409 - Pimasertib	NCT01390818(1)	
	PI3K-mTOR inhibitor – MEK inhibitor	BEZ235 - MEK162	NCT01337765(1)	
	MEK inhibitor - SRC inhibitor	Selumetinib - Dasatinib	NCT00780676(2)	
	mTOR inhibitor – EGFR inhibitor	Temsirolimus - Neratinib	NCT01111825(2)	
	mTOR inhibitor-+ NY-ESO-1 vaccine	Sirolimus - DEC-205-NY-ESO-1 vaccine	NCT01522820(1)	
	VEGF inhibitor - Anti-PDL1	Bevacizumab - MPDL3280A	NCT01633970(1)	

Many clinical trials are actively investigating new drugs in breast cancers. All ongoing trial could enroll TNBCs; most do not have inclusion criteria restricted to TNBCs (Table 2). We also report here the relevant trials that illustrated our classification. Trials enrolling:

*only BRCA1 or 2 mutation carriers BCs;

**ER-positive BCs;.

#AR-positive BCs;

##HER2-negative breast cancers.

†Ongoing trials evaluating targeted therapy in combination with platinum-based regimen.

Abbreviations: AR, androgen receptor; (B), biomarkers trial; BC, breast cancer; CHT, chemotherapy; CTLA-4, cytotoxic-T-lymphocyte-antigen-4; DLL, delta-like ligand; EGFR, epidermal growth factor receptor; FGFR, fibroblast growth factor receptor; FRZ, Frizzle receptor; HER2, human epidermal growth factor receptor 2; HSP, heat-shock protein; IAP, inhibitor of apoptosis protein; LT, lymphocyte T; mAb, monoclonal antibody; MUC1, mucin-1; ORR, objective response rate; OS, overall survival; pCR, pathological response rate; PD-1, programmed-death-1; PD-L1, programmed-death-ligand-1; PFS, progression-free survival; SARM, selective androgen receptor modulator; SMO, smoothened; TGFβ, transforming growth factor β; TKI, tyrosine-kinase inhibitor; VEGF, vascular endothelial growth factor receptor

REFERENCES IN SUPPLEMENTARY TABLE 1 [1–12]

1. Kaufman B, Shapira-Frommer R, Schmutzler RK, Audeh MW, Friedlander M, Balmaña J, Mitchell G, Fried G, Stemmer SM, Hubert A, Rosengarten O, Steiner M, Loman N, Bowen K, Fielding A, Domchek SM. Olaparib monotherapy in patients with advanced cancer and a germline BRCA1/2 mutation. J Clin Oncol. 2015 Jan 20; 33:244–50.
2. Vonderheide RH, LoRusso PM, Khalil M, Gartner EM, Khaira D, Soulieres D, Dorazio P, Trosko JA, Rüter J, Mariani GL, Usari T, Domchek SM. Tremelimumab in Combination with Exemestane in Patients with Advanced Breast Cancer and Treatment-Associated Modulation of Inducible Costimulator Expression on Patient T Cells. Clin Cancer Res. 2010 Jul 1; 16:3485–94.
3. Diab A, McArthur HL, Solomon SB, Sacchini V, Comstock C, Maybody M, Durack JC, Blum B, Yuan J, Patil S, Neville DA, Comen EA, Morris EA, Kotin A, Brogi E, Morrow M, Allison JP, Hudis CA, Norton L, Wolchok JD. A pilot study of preoperative (Pre-op), single-dose ipilimumab (Ipi) and/or cryoablation (Cryo) in women (pts) with early-stage/resectable breast cancer (ESBC). J Clin Oncol [Internet]. 2014; [cited 2014 Jun 6]; 32:5s. Available from: http://meetinglibrary.asco.org/content/132420-144.
4. Miles D, Roché H, Martin M, Perren TJ, Cameron DA, Glaspy J, Dodwell D, Parker J, Mayordomo J, Tres A, Murray JL, Ibrahim NK. . Theratope® Study GroupPhase III multicenter clinical trial of the sialyl-TN (STn)-keyhole limpet hemocyanin (KLH) vaccine for metastatic breast cancer. Oncologist. 2011; 16:1092–100.
5. Gartner EM, Silverman P, Simon M, Flaherty L, Abrams J, Ivy P, Lorusso PM. A phase II study of 17-allylamino-17-demethoxygeldanamycin in metastatic or locally advanced, unresectable breast cancer. Breast Cancer Res Treat. 2012 Feb; 131:933–7.
6. Gianni L, Lladó A, Bianchi G, Cortes J, Kellokumpu-Lehtinen P-L, Cameron DA, Miles D, Salvagni S, Wardley A, Goeminne J-C, Hersberger V, Baselga J. Open-label, phase II, multicenter, randomized study of the efficacy and safety of two dose levels of Pertuzumab, a human epidermal growth factor receptor 2 dimerization inhibitor, in patients with human epidermal growth factor receptor 2-negative metastatic breast cancer. J Clin Oncol. 2010 Mar 1; 28:1131–7.
7. Mittendorf EA, Clifton GT, Holmes JP, Clive KS, Patil R, Benavides LC, Gates JD, Sears AK, Stojadinovic A, Ponniah S, Peoples GE. Clinical trial results of the HER-2/neu (E75) vaccine to prevent breast cancer recurrence in high-risk patients: from US Military Cancer Institute Clinical Trials Group Study I-01 and I-02. Cancer. 2012 May 15; 118:2594–602.
8. Baselga J, Segalla JGM, Roché H, Del Giglio A, Pinczowski H, Ciruelos EM, Filho SC, Gómez P, Van Eyll B, Bermejo B, Llombart A, Garicochea B, Durán MÁC, Hoff PMG, Espié M, de Moraes AAJG, Ribeiro RA, Mathias C, Gil Gil M, Ojeda B, Morales J, Kwon Ro S, Li S, Costa F. Sorafenib in combination with capecitabine: an oral regimen for patients with HER2-negative locally advanced or metastatic breast cancer. J Clin Oncol. 2012 May 1; 30:1484–91.
9. Taylor SK, Chia S, Dent S, Clemons M, Agulnik M, Grenci P, Wang L, Oza AM, Ivy P, Pritchard KI, Leighl NB. A phase II study of pazopanib in patients with recurrent or metastatic invasive breast carcinoma: a trial of the Princess Margaret Hospital phase II consortium. Oncologist. 2010; 15:810–8.
10. Bergh J, Bondarenko IM, Lichinitser MR, Liljegren A, Greil R, Voytko NL, Makhson AN, Cortes J, Lortholary A, Bischoff J, Chan A, Delaloge S, Huang X, Kern KA, Giorgetti C. First-line treatment of advanced breast cancer with sunitinib in combination with docetaxel versus docetaxel alone: results of a prospective, randomized phase III study. J Clin Oncol. 2012 Mar 20; 30:921–9.
11. Winer EP, Tolaney S, Nechushtan H, Berger R, Kurzrock R, Ron I, Schoffski P. Activity of cabozantinib (XL184) in metastatic breast cancer (MBC): Results from a phase II randomized discontinuation trial (RDT). J Clin Oncol. 2012; 30.
12. André F, Bachelot T, Campone M, Dalenc F, Perez-Garcia JM, Hurvitz SA, Turner N, Rugo H, Smith JW, Deudon S, Shi M, Zhang Y, Kay A, Porta DG, Yovine A, Baselga J. Targeting FGFR with Dovitinib (TKI258): Preclinical and Clinical Data in Breast Cancer. Clin Cancer Res. 2013 Jul 1;19:3693–702.
